# Simultaneous Immunoassay Analysis of Plasma IL-6 and TNF-α on a Microchip

**DOI:** 10.1371/journal.pone.0053620

**Published:** 2013-01-09

**Authors:** Kaori Abe, Yoshiko Hashimoto, Shouki Yatsushiro, Shohei Yamamura, Mika Bando, Yuka Hiroshima, Jun-ichi Kido, Masato Tanaka, Yasuo Shinohara, Toshihiko Ooie, Yoshinobu Baba, Masatoshi Kataoka

**Affiliations:** 1 Health Research Institute, National Institute of Advanced Industrial Science and Technology (AIST), Takamatsu, Japan; 2 Department of Periodontology and Endodontology, Oral and Maxillofacial Dentistry, Division of Medico-Dental Dynamics and Reconstruction, Institute of Health Biosciences, University of Tokushima, Tokushima, Japan; 3 Faculty of Pharmaceutical Sciences, University of Tokushima, Tokushima, Japan; 4 Institute for Genome Research, University of Tokushima, Tokushima, Japan; 5 Department of Applied Chemistry, Graduate School of Engineering, Nagoya University, Nagoya, Japan; University of Strathclyde, United Kingdom

## Abstract

Sandwich enzyme-linked immunosorbant assay (ELISA) using a 96-well plate is frequently employed for clinical diagnosis, but is time-and sample-consuming. To overcome these drawbacks, we performed a sandwich ELISA on a microchip. The microchip was made of cyclic olefin copolymer with 4 straight microchannels. For the construction of the sandwich ELISA for interleukin-6 (IL-6) or tumor necrosis factor-α (TNF-α), we used a piezoelectric inkjet printing system for the deposition and fixation of the 1st anti-IL-6 antibody or 1st anti-TNF-α antibody on the surface of the each microchannel. After the infusion of 2 µl of sample to the microchannel and a 20 min incubation, 2 µl of biotinylated 2nd antibody for either antigen was infused and a 10 min incubation. Then 2 µl of avidin-horseradish peroxidase was infused; and after a 5 min incubation, the substrate for peroxidase was infused, and the luminescence intensity was measured. Calibration curves were obtained between the concentration and luminescence intensity over the range of 0 to 32 pg/ml (IL-6: R^2^ = 0.9994, TNF-α: R^2^ = 0.9977), and the detection limit for each protein was 0.28 pg/ml and 0.46 pg/ml, respectively. Blood IL-6 and TNF-α concentrations of 5 subjects estimated from the microchip data were compared with results obtained by the conventional method, good correlations were observed between the methods according to linear regression analysis (IL-6: R^2^ = 0.9954, TNF-α: R^2^ = 0.9928). The reproducibility of the presented assay for the determination of the blood IL-6 and TNF-α concentration was comparable to that obtained with the 96-well plate. Simultaneous detection of blood IL-6 and TNF-α was possible by the deposition and fixation of each 1st antibody on the surface of a separate microchannel. This assay enabled us to determine simultaneously blood IL-6 and TNF-α with accuracy, satisfactory sensitivity, time saving ability, and low consumption of sample and reagents, and will be applicable to clinic diagnosis.

## Introduction

The expression of inflammatory cytokines including interleukin-6 (IL-6) and tumor necrosis factor-α (TNF-α) has been associated with not only acute inflammation but also a number of chronic conditions associated with aging, such as cardiovascular disease, diabetes, physical disabilities and cognitive decline [1]. Plasma concentrations of these cytokines at reference values are several pg/ml, and they increase in several of the diseases mentioned above. The availability of methods to measure these cytokines with high sensitivity and specificity is critically important. The double antibody sandwich technique, the so-called sandwich enzyme-linked immunosorbant assay (ELISA), is frequently used for antigen measurement [2], [3]. This assay is specifically useful for detecting a specific antigen when only a small amount of it is available and purified antigen is unavailable. In the conventional sandwich ELISA system, disposable 96-well microtitration plates are frequently employed to detect these inflammatory cytokines in blood. Typically 50 µl aliquots of capture antibody (1st antibody), enzyme conjugated antibody (2nd antibody) to detect antigen, and 50 µl aliquots of antigen solution are introduced into the reaction wells of 96-well microtitration plates [4]. Furthermore, this method is time-consuming, for the capture of antigen by the 1st antibody and the determination of the antigen concentration by the 2nd antibody each requires 2 hr or more. So, the conventional sandwich ELISA for the determination of plasma cytokines is not suitable for rapid diagnosis.

The analysis of biomarkers at a location near the patient, which analysis is called point of care testing (POCT), is a continuously expanding trend in the practice of laboratory diagnosis [5]; and it is preferred when test results are needed more rapidly than is feasible by the use of conventional testing procedures [6]–[9]. Several types of microchips have been developed for chemical and biological analyses [10]–[13], and several types of immunoassay on a microchip have been applied practically in POCT [14]–[22]. Such analysis using microchips has many advantages such as high efficiency, time-saving ability, ease of operation, low consumption of samples and reagents, and easy integration and automation. Antibody adsorbed onto beads-packed in microchannels has often been employed for the sandwich ELISA [17], [19]–[22]. For these immunoassays, elaborate microscopic dam-like structures (a few score or so of µm scale gaps) in the microchannel are needed to stop these microbeads in suspension from flowing throughout the microchannel. In contrast, inkjet printing allows for the straightforward deposition of biological materials on the assay surface; and inkjet printing systems to deposit and immobilize an antibody on a nylon membrane have been used for immunoassay application [23]. Recently, we developed the sandwich ELISA performed in a microchannel for quantitative analysis of carboxyterminal propeptide of type I procollagen (PICP) in the blood; and piezoelectric inkjet printing was employed for deposition and fixation of the 1st antibody on the microchannel surface [24].

In the present study, we demonstrated the potential of the sandwich ELISA on a microchip, with the 1st antibody deposited by piezoelectric inkjet printing for the quantitative analysis of IL-6 and TNF-α which concentrations are very low in the blood of healthy individuals (pg/ml levels). Furthermore, simultaneous quantitative detection of IL-6 and TNF-α was performed by using the sandwich ELISA in independent microchannels on a microchip.

## Methods

### Principle

**Figure 1:**
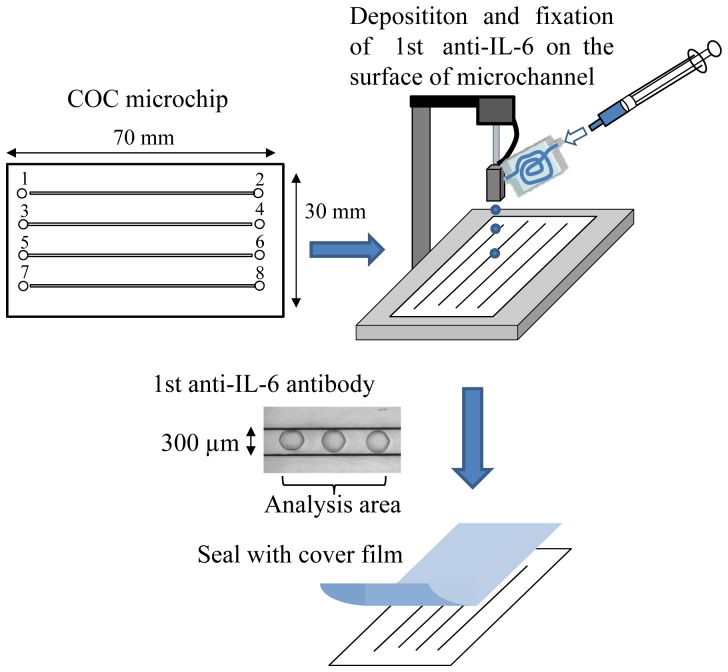
Schematic process for the detection of IL-6 and/or TNF-α on a microchip with deposition and fixation of 1st antibody by piezoelectric ink printing. After fixation of antibody on the surface of the microchannel and sealing with a cover film, blocking solution was infused into each microchannel from the reservoir (wells 1, 3, 5, and 7) by use of a pipetman.

The principle of the sandwich ELISA on a microchip was described in our earlier report [24]. A schematic illustration of the sandwich ELISA on a microchip for the determination of plasma IL-6 and TNF-α is shown in Fig. 1. The microchip (BS-X2321, SUMITOMO BAKELITE Co., Ltd, Tokyo, Japan) was made of cyclic olefin copolymer (COC) and was 30 mm long and 70 mm wide. The channels in the microchip were straight, 300 µm wide, and 100 µm deep. The COC microchip was fabricated by injection molding with a nickel mold, and the microchip surface was treated with polymer solution containing p-nitrophenyl ester, which binds to amino groups in proteins for 1st antibody fixation on the microchannel surface. This type of microchip was used in this immunoassay for the detection of plasma IL-6 or TNF-α. Four samples could be analyzed simultaneously on the microchip. A Pulseinjector (Cluster Technology Co., Ltd., Osaka, Japan), which is a piezoelectric inkjet printing system, was employed for the deposition and fixation of the 1st antibody on the microchannel. The drive waveform B with a triangular pulse (width 100 µm) was employed to provide the volume of the droplet, and the droplets were ejected at a frequency of 20 Hz and a jetting voltage of 8 V. The volume of a single discharged droplet of 1st antibody was 120 pl, and 12 droplets were discharged onto the surface of the microchannel to fix the maximum volume of the 1st antibody, as shown in Fig. 1. After the deposition and fixation of the 1st anti-IL-6 or anti-TNF-α antibody on the surface of the microchannel, the microchip surface was sealed with a cover film made of polymethylmethacrylate having a 47 µm thickness and a 33 µm adhesion layer (TOYO INK MFG. CO., LTD. Tokyo, Japan). All samples and reagents for the sandwich ELISA assay were infused from reservoirs with a diameter of 1.0 mm (wells 1, 3, 5, and 7) into the microchannels by a pipette. The reservoirs on the other side of the microchip were used as a waste reservoir (wells 2, 4, 6, and 8).

### Reagents and Analytical Procedure

Human IL-6 ELISA MAX Deluxe Sets were purchased from BioLegnd (San Diego, CA, USA) and employed for the determination of the plasma concentration of IL-6 with a microtitration plate as the conventional method according to the supplied instructions. Briefly, 100 µl of Human IL-6 ELISA MAX™ Capture Antibody (2 µg/ml) was coated on a 96-well microtitration plate overnight at 4°C as 1st antibody, and then blocked with 200 µl of 1X Assay Diluent for 1 hr at room temperature. Then, 100 µl of standard sample or plasma sample was added to each well and incubated for 2 hr at room temperature. To construct a calibration curve we diluted Human IL-6 Standard with 1X Assay diluent A to 32, 16, 8, 4, and 2 pg/ml and used them as standard samples. Next, 100 µl of biotinylated Human IL-6 ELISA MAX™ Detection Antibody as 2nd antibody was added to each well, followed by a 1 hr incubation at room temperature. Thereafter, 100 µl of avidin-horseradish peroxidase (HRP) was subsequently added; and incubation was continued for 30 min at room temperature. After washing of the microtitration plate with wash buffer, 100 µl of substrate solution for peroxidase was added to each well; and the plate was then incubated for 30 min at room temperature. Finally, stop solution was added to each well; and the absorbance of the solution was then measured at 450 nm by using a microplate reader (Tecan Austria GmbH, Salzburg, Austria). The plasma concentration of IL-6 was calculated from the calibration curve and expressed as pg/ml.

Human TNF-α antibody (MAB610) as 1st antibody, biotinylated anti-human TNF-α/TNFSF1A antibody (BAF210) as 2nd antibody, and recombinant human TNF-α (210-TA) as standard were purchased from R&D Systems Inc. (Minneapolis, MN, USA) and employed for the determination of the plasma concentration of TNF-α with a microtitration plate as the conventional method. Briefly, 100 µl of 1st anti-TNF-α antibody was coated onto the wells of a 96-well microtitration plate, after which 100 µl of standard sample or plasma sample was added to each well. Following a 2 hr incubation at room temperature, 100 µl of 2nd anti-TNF-α antibody was added to each well; and incubation was carried out for 2 hr at room temperature. Finally, 100 µl of Streptoavidin-HRP was subsequently added to each well; and after 20 min, 100 µl of substrate solution was then added to the well. To construct a calibration curve, we diluted TNF-α with 1X Assay diluent to 32, 16, 8, 4, and 2 pg/ml and used them as standard samples. The plasma concentration of TNF-α was calculated from the calibration curve and expressed as pg/ml.

### Analysis of IL-6 and TNF-α on the Microchip

For the sandwich ELISA assay on the microchip, we used the same monoclonal anti-IL-6 antibodies and anti-TNF-α antibodies that were used in the 96-well microtitration plates. For the deposition and fixation of 1 mg/ml 1st anti-IL-6 antibody and 1st anti-TNF-α antibody in spotting buffer (SUMITOMO BAKELITE Co., Ltd) on the surface of the microchannel, a piezoelectric inkjet printing system (Cluster Technology Co., Ltd. Osaka, Japan) was employed. A jetting voltage of 8 V for antibody “inks” was used in this study. The volume of the drop ejected from the inkjet nozzle was 120 pl; and 12 drops were spotted onto the microchannel surface, 3 of which are depicted in Figure 1. The microchip was then incubated for 4 hr at room temperature. Spots were round with a 250 µm diameter. After sealing of the microchip surface with the cover film, BlockAce (DS Pharma Biomedical Co., Ltd., Osaka, Japan), a blocking solution was infused into each microchannel; and the microchip was left undisturbed overnight at 4°C. Washing of the microchannels was performed by the infusion of 0.25% (v/v) Triton X-100 in PBS into each microchannel. Then 2 µl of sample was infused into each microchannel, followed by incubation at room temperature for 20 min. Samples were prepared according to the conventional method described above. To construct a calibration curve and to examine the reproducibility in a single channel or different channels, we diluted standards with 1X Assay Diluent to 32, 16, 8, 4, and 2 pg/ml; and they were used as samples. After washing of the microchannels with 0.25% Triton X-100 in PBS, 2 µl of biotinylated anti-human IL-6 antibody or anti-TNF-α antibody as 2nd antibody was infused into the microchannels; and the microchip was incubated for 10 min at room temperature. After the microchannels had been washed with 0.25% Triton X-100 in PBS, 2 µl of avidin- HRP was infused into each microchannel; and the microchip was incubated for 5 min at room temperature. After washing of the microchannels with 0.25% Triton X-100 in PBS, 2 µl of SuperSignal West Femto Chemiluminescent Substrate (Thermo Scientific, Rockford, IL), which is a chemiluminescent substrate for detection of peroxidase activity, was infused into each microchannel. The integration value of luminescence intensity of each spot was measured with an ImageQuant LAS4000 (GE Healthcare UK Ltd, Buckinghamshire, England) which has a resolution of up to 70 µm and analyzed by using an ImageQuant TL software (GE Healthcare UK Ltd).

### Blood Sample Preparation

Peripheral venous blood samples were collected from healthy subjects by standard venipuncture. All blood samples were collected into vacuum blood-collecting tubes containing EDTA-2Na (VENOJECT II, TERUMO, Tokyo, Japan), and the plasma samples were obtained by centrifugation at 1450×g for 10 min. The plasma was then stored at −80°C until used for determination of protein levels.

### Ethics

This study was approved by the Institutional Review Board of the National Institute of Advanced Industrial Science and Technology for the use of human derivatives for biomedical research, and by the Ethics Committee of the University of Tokushima. All subjects provided written informed consent for the collection of samples and subsequent analysis.

## Results

### Sandwich ELISA on a Microchip

**Figure 2:**
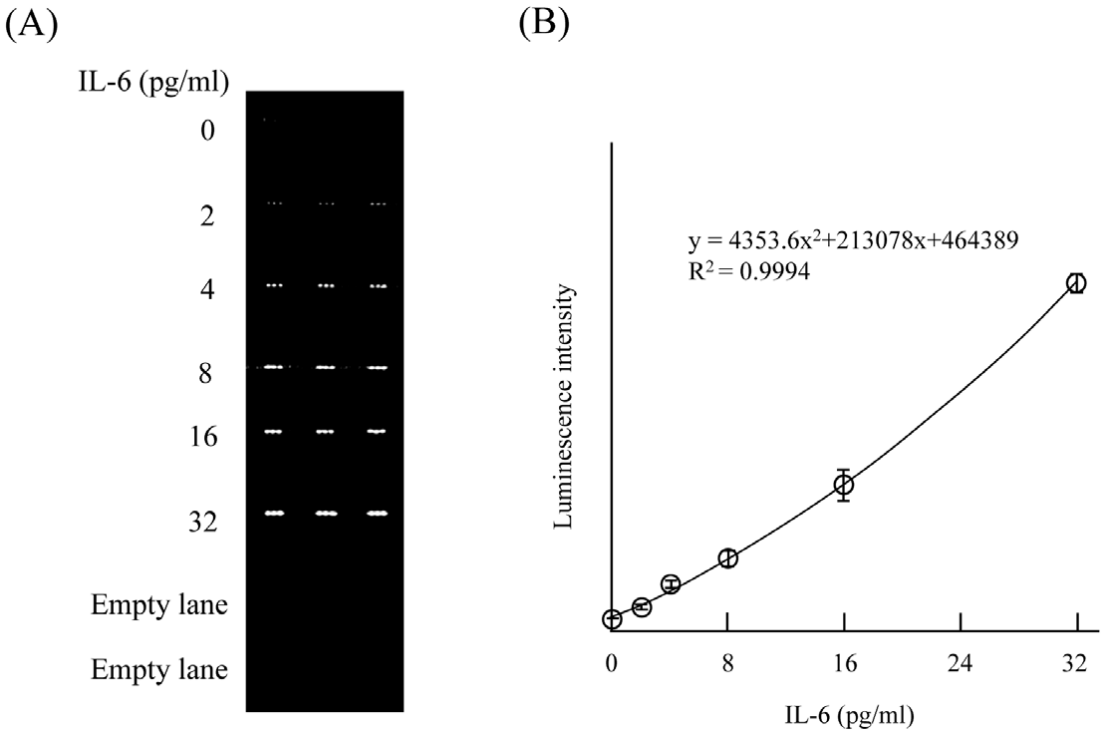
Detection of purified IL-6 by sandwich ELISA on a microchip. Three deposits of 1st antibody were made into each microchannel, and 0 to 32 pg/ml IL-6, biotin-conjugated 2nd antibody, avidin-conjugated HRP solution were then infused, allowing triplicate determinations to be made in one channel. (A) The luminescence increased in an IL-6 concentration-dependent manner. (B) Standard curve for the IL-6 concentration versus luminescence intensity obtained by the assay.

**Figure 3:**
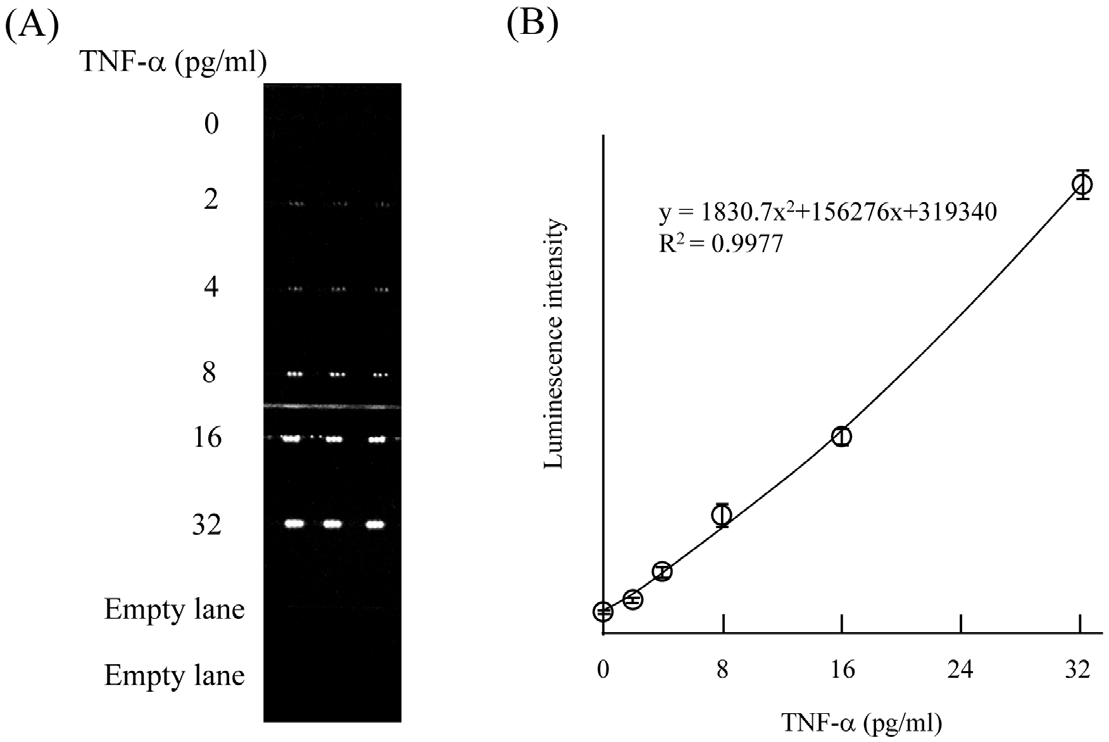
Detection of purified TNF-α by sandwich ELISA on a microchip. Three deposits of 1st antibody were made into each microchannel, and 0 to 32 pg/ml TNF-α, biotin-conjugated 2nd antibody, avidin-conjugated HRP solution were then infused, allowing triplicate determinations to be made in 1 channel. (A) The luminescence increased in a TNF-α concentration-dependent manner. (B) Standard curve for the TNF-α concentration versus luminescence intensity obtained by the assay.

In the present study, we developed sandwich ELISAs on a COC microchip by depositing and fixing the 1st anti-IL-6 antibody and/or 1st anti-TNF-α antibody on the surface of the microchannels with a piezoelectric inkjet printing system (Fig. 1). In both cases, the luminescence intensity increased in a time-dependent manner after the sample was introduced into the microchannel, and linear relationships were obtained until 40 min (data not shown). Therefore, every reaction time was fixed at 20 min in the present study. Although the luminescence intensities for both IL-6 and TNF-α increased in a concentration-dependent manner (Fig. 2A, Fig. 3A), the luminescence intensities at 2 pg/ml were very weak for both proteins. As the luminescence intensity depends on the area of the fixed 1st antibody (data not shown), we employed the sum of luminescence intensity of 3 spots as 1 analysis area in all experiments. Reproducibility of the determination of the IL-6 or TNF-α concentration in 1 channel and different channels was examined. As shown in Table 1, slight variation of the luminescence intensity of the 3 analysis areas in 1 channel for 0, 2, 4, 8, 16, and 32 pg/ml IL-6 was observed, with the relative standard deviation (RSD) for each concentration being 8.8, 4.8, 6.9, 6.8, 7.0, and 7.3%, respectively. Slight variation of the luminescence intensity of 3 analysis areas in 1 channel for 0, 2, 4, 8, 16, and 32 pg/ml TNF-α was also observed, with the RSD for each concentration being 6.2, 5.7, 6.8, 9.9, 3.5, and 3.2%, respectively (Table 2). These result indicated the possibility of using a single channel with multi spotting of antibody on the microchannel for duplicate, triplicate, quadruplicate or more determinations by using only 1 channel. The RSDs of the luminescence intensity in 3 different channels for 0, 2, 4, 8, 16, and 32 pg/ml IL-6 were 5.4, 3.2, 5.9, 8.0, 8.2, and 9.9%, respectively (Table 3). A similar tendency was also observed for TNF-α, the RSDs for 0, 2, 4, 8, 16, and 32 pg/ml TNF-α being 4.1, 4.8, 7.2, 7.6, 8.5, and 6.1%, respectively (Table 4). These results indicated good reproducibility of luminescence intensity induced by the antigen-antibody reaction even in different microchannels.

**Table 1:** Reproducibility of luminescence intensity of IL-6 in 1 channel.

Number of analysisareas	IL-6 (pg/ml)					
	0	2	4	8	16	32
1	306534	606067	1131618	2567535	5119245	10111860
2	364432	651499	1168431	2319906	5669754	10845244
3	348595	664145	1020849	2260658	5867182	11705519
Average	339854	640570	1106966	2382700	5552060	10887541
RSD (%)	8.8	4.8	6.9	6.8	7.0	7.3

**Table 2:** Reproducibility of luminescence intensity of TNF-α in 1 channel

Number of analysisareas	TNF-α (pg/ml)					
	0	2	4	8	16	32
1	306619	485013	1064842	1957041	3030006	7080520
2	343273	533984	942603	2045302	3236647	7085038
3	340801	538138	955622	1686031	3193599	7481575
Average	330231	519045	987689	1896125	3153417	7215711
RSD (%)	6.2	5.7	6.8	9.9	3.5	3.2

**Table 3:** Reproducibility of luminescence intensity of IL-6 in 3 different channels.

Channel number	IL-6 (pg/ml)					
	0	2	4	8	16	32
1	339854	638608	998876	2721310	5019289	9226740
2	375331	640570	1106966	2372981	5919242	9213155
3	346090	680632	1107943	2382700	5552060	10887541
Average	353758	658826	1071261	2492330	5496864	9775812
RSD (%)	5.4	3.24	5.9	8.0	8.2	9.9

**Table 4:** Reproducibility of luminescence intensity of TNF-α in 3 different channels.

Channel number	TNF-α (pg/ml)					
	0	2	4	8	16	32
1	344089	536864	983281	1745975	2995462	6556234
2	335895	525924	1002687	2030188	3142440	7113636
3	363595	489803	1122053	1941099	3526220	7404974
Average	347860	517530	1036007	1905754	3221374	7024948
RSD (%)	4.1	4.8	7.3	7.6	8.5	6.1

### Determination of Plasma IL-6 and TNF-α

**Figure 4:**
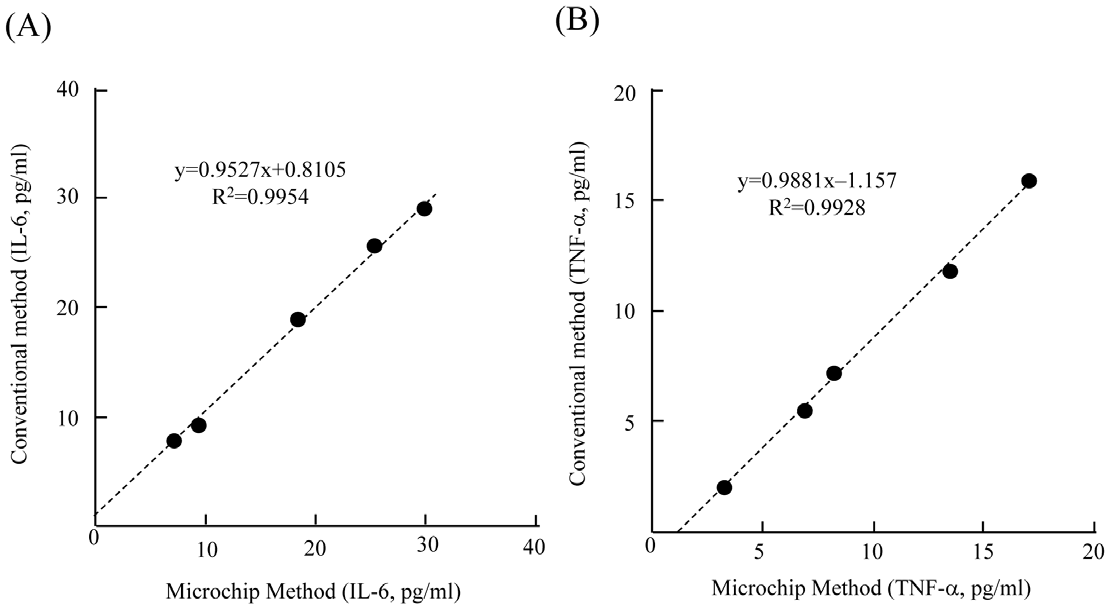
Comparative analysis of the values obtained with the microchip method and the conventional microtitration plate method. Linear regression analysis was used. (A) IL-6 (B) TNF-α.

Quantitative analysis of plasma IL-6 or TNF-α concentrations was performed next. The relationship between the concentrations of purified IL-6 or TNF-α in the range of 0–32 pg/ml and the luminescence intensity derived from biotin-labeled 2nd antibody and avidin-labeled HRP was examined (Fig. 2 and 3). Three analysis areas in 1 microchannel were used for triplicate determinations, and purified IL-6 or TNF-α solution (0 to 32 pg/ml), biotinylated 2nd antibody, and avidin-HRP were infused into each microchannel. The mean (± standard deviation [SD]) of the luminescence intensity for the 3 different analysis areas at each concentration of purified IL-6 or TNF-α was obtained and plotted against the dose, between the range of 0–32 pg/ml IL-6 or TNF-α. The calibration curves showed that the luminescence intensity increased with increasing IL-6 (Fig. 2B) or TNF-α (Fig. 3B) concentration. The limit of detection (LOD) for IL-6 was 0.28 pg/ml, which gave a signal at 3 SDs above the background [25]. The LOD for TNF-α was 0.46 pg/ml, which gave a signal at 3 SDs above the background. These ranges are sufficient for clinical estimation of IL-6 and TNF-α concentrations in the blood [26]. These data thus strongly indicate that this sandwich ELISA performed on a microchip with the aid of piezoelectric printing technology is suitable for the determination of plasma IL-6 and TNF-α concentrations. Plasma IL-6 and TNF-α concentrations in 5 subjects were then calculated by reference to the calibration curve generated from data obtained from the sandwich ELISA on a microchip. Each estimated plasma IL-6 or TNF-α concentration was compared with the results obtained by using the respective 96-well microtitration plate. As shown in Fig. 4, linear regression analysis of IL-6 and TNF-α concentrations obtained by both methods revealed a significant relationship (IL-6 : R^2^ = 0.9954, TNF-α : R^2^ = 0.9928). These results indicated that accurate determination of plasma IL-6 and TNF-α could be achieved by use of our new method. Three different samples were analyzed repeatedly within 1 day or for several days for their IL-6 and TNF-α concentration (Tables 5 and 6). The within-day (n = 3) reproducibility of the determination of IL-6 in plasma samples was 5.2–7.3%; and the between-days (n = 3) one, 4.3–9.2% (Table 5). The within-day (n = 3) reproducibility of the determination of TNF-α in plasma samples was 2.3–5.9%; and the between-days (n = 3) one, 7.9–9.8% (Table 6). According to the documentation supplied in the IL-6 ELISA kit (BioLegend), reproducibilities of within-day and between-days were 4.3–11.3% and 4.5–13%, respectively, for 2 different subjects when the conventional microtitration plate was used. As described in the Methods section, we developed the sandwich ELISA with a microtitration plate as the conventional method for the determination of plasma TNF-α. The reproducibilities of this assay were calculated. The reproducibilities of within-day and between-days were 7.5–9.7% and 8.0–10.8%, respectively, for 3 different subjects (data not shown). Thus the reproducibility of the sandwich ELISA on a microchip for the determination of the plasma IL-6 and TNF-α concentration was quite comparable to that obtained with the conventional 96-well microtitration plate.

**Table 5:** Reproducibility of microchip method for the determination of IL-6 in plasma samples.

Sample	IL-6	
	Mean ± SD, pg/ml	RSD, %
Within day, n = 3		
1	12.2±0.7	5.7
2	19.1±1.4	7.3
3	27.7±1.44	5.2
Between days, n = 3		
4	6.1±0.3	4.3
5	9.3±0.7	7.2
6	18.9±1.7	9.2

**Table 6:** Reproducibility of microchip method for the determination of TNF-α in plasma samples

Sample	TNF-α	
	Mean ± SD, pg/ml	RSD, %
Within day, n = 3		
1	7.0±0.4	5.9
2	11.6±0.7	5.9
3	19.1±0.4	2.3
Between days, n = 3		
4	6.7±0.5	8.0
5	12.7±1.0	7.9
6	16.0±1.6	9.8

**Figure 5:**
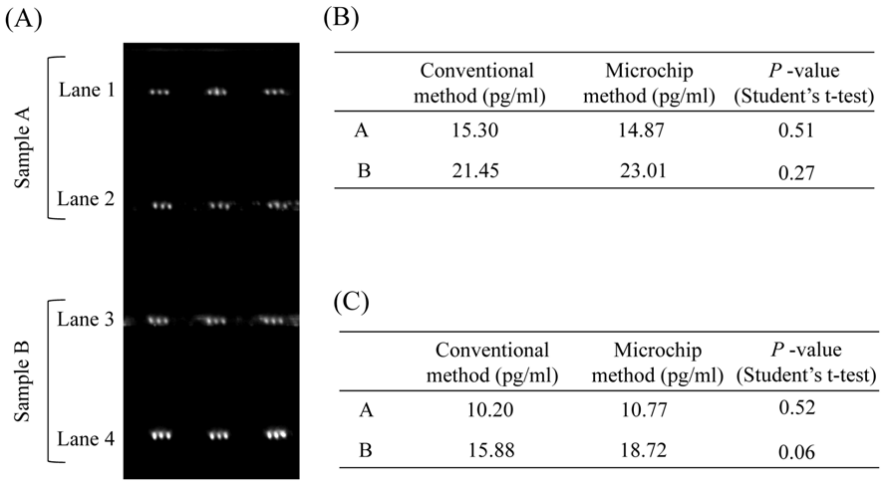
Images of plasma IL-6 and TNF-α detected by sandwich ELISA on a microchip. (A) The detection image of plasma IL-6 and TNF-α on a microchip. 1st anti-IL-6 antibody was fixed on the surface of microchannels 1 and 3; and 1st anti- TNF-α antibody, on that of microchannels 2 and 4. (B) Comparison of the plasma IL-6 concentration obtained with the microchip method and the conventional microtitration plate method. (C) Comparison of the plasma TNF-α concentrations obtained with the microchip method and the conventional microtitration plate method.

Next, we tested to see if we could determine the concentrations of plasma IL-6 and TNF-α simultaneously in separate microchannels on a microchip (Fig. 5). Anti-IL-6 antibody was deposited and fixed in 2 microchannels; and anti- TNF-α antibody, in the other 2 microchannels. The plasma samples were infused into each microchannel. Then, the concentrations of IL-6 and TNF-α in plasma from 2 subjects were determined as described above. Significance differences in estimated plasma concentrations of IL-6 or TNF-α by the conventional method and microchip method were examined by performing Student’s *t*-test. There were no significance differences between the 2 methods for either IL-6 (Fig. 5B) or TNF-α (Fig. 5C; *p*>0.05). These results indicated that simultaneous quantitative detection of plasma IL-6 and TNF-α could be performed on a microchip.

## Discussion

We employed an inkjet printing system to develop the quantitative sandwich ELISA for measuring plasma IL-6 and TNF-α in the microchannel, because it has been shown to deliver small reagent volumes with precise alignment and high reproducibility [27]. Although we had earlier developed a quantitative sandwich ELISA to determine serum PICP by using a similar inkjet printing system with high accuracy, high sensitivity, time-saving ability, and low consumption of sample and reagents, its detection limit was 4.7 ng/ml [24]. In the PICP detection on a microchip, high sensitivity and time-saving detection could be obtained just by using the same concentrations of antibodies as used in 96-well plates. When the same concentrations of antibodies and immunoassay time (Total 3.5 hr: 1st antibody for 2 hr, 2nd antibody for 1 hr, Avidin-HRP for 30 min) for the detection of IL-6 and TNF-α in 96-well plates were used for the microchip, the detection limits of both proteins were around 100 pg/ml (data not shown). Reference values of plasma concentrations of IL-6 and TNF-α are several pg/ml. So, remarkably high sensitivity and rapid detection would be necessary for the detection of plasma IL-6 and/or TNF-α in the microchannel. We employed highly concentrated 1st antibodies for IL-6 (500 times higher than that for the conventional method) and/or TNF-α (125 times higher than that for the conventional method) were employed for the deposition and fixation on the surface of the microchamber for the actualization of highly sensitive detection and reduced immunoassay time. By using highly concentrated 1st antibody for IL-6 or TNF-α detection, equal to or higher sensitivity and a remarkable reduction in immunoassay time were obtained for the detection of both proteins (210 min for IL-6 or 260 min for TNF-α by 96-well microtitration plate versus 35 min by microchip method). Although we could not compare the consumption of 1st anti-IL-6 antibody between the microtitration plate and the microchip because the concentration of the 1st anti-IL-6 antibody used for the microtitration plate was not known, low consumption of 1st anti-TNF-α antibody (800 ng/well for 96-well microtitration plate versus 4.3 ng/analysis area for microchip) was observed. Furthermore, the use of a microchip has considerable advantages over the microtitration plate for the analysis of either IL-6 or TNF-α: low consumption of plasma, 2nd antibody, and avidin-HRP (100 µl of each/well for 96 well-microtitration plate versus 2 µl/microchannel), and ease of operation, i.e., the use of just 1 pipetman for infusion of each solution into the microchannel.

Microchip-based multianalyte assay systems using a sandwich ELISA had already been reported earlier. In these assays, 1st-antibody-conjugated beads were introduced into the microchip and employed for the detection of several biomarkers [25], [28]. Christodoulides et al. reported the simultaneous detection of 1 to 10,000 ng/ml C-reactive protein and 1000 ng/ml IL-6 in human serum samples [28], but the quantitative detection of IL-6 was not shown. Ikami et al. had developed the immuno-pillar chip, having hydrogel pillars, fabricated inside a microchannel, with many antibody molecules immobilized onto polystyrene beads [25]. Simultaneous and quantitative analysis of C-reactive protein, α-fetoprotein, and prostatic-specific antigen were performed in microchannels in which the pillars were made with 3 kinds of beads bearing immobilized antibodies specific for these 3 kinds of proteins. The detection limit of each of the biomarkers was about 100 pg/ml. We demonstrated the simultaneous quantitative analysis of plasma IL-6 and TNF-α on a microchip by using an individual microchannel for each cytokine with a detection limit sufficiently low for clinical estimation. Although we cannot compare wholly the sensitivities because of the differences in the kinds of antibodies and the immunoassay times, we were able to develop a highly sensitive sandwich ELISA for these cytokines on a microchip. The principal reason for the high sensitivity of this method for these cytokines may be the use of extremely highly concentrated 1st antibody for deposition and fixation on a microchannel created by an inkjet printing system. In the immunoassay using the antibody-conjugated beads, there is a limited number of antibody-conjugated beads that can be introduced into the microchannel. We tried to deposit and fix 1st anti-IL-6 and anti-TNF-α antibodies on separate parts of the surface of 1 microchannel to allow multianalyte assay of cytokines in a single microchannel. The concentrations of both cytokines were estimated to have been significantly higher than their actual concentrations (data not shown). We do not presently know the reason for these inaccurate results. So, the individual microchannel on a microchip was employed for each cytokine for quantitative multianalyte assay.

In conclusion, we performed simultaneous analysis of IL-6 and TNF-α in human plasma with high accuracy and reproducibility. Furthermore, this assay also offers other considerable advantages, including low consumption of sample and reagents, reduced assay time, and ease of operation. This assay is thus applicable to POCT.
